# 

**Published:** 1907-10-26

**Authors:** 


### CONTENTS.

| | PAGE |
|---|---|
| **On Metastases in Malignant Disease** | 77 |
| **Some Medical Aspects of the Peace Conference** | 78 |
| **Annotations—** | |
| The Calmette Serum Reaction in Tuberculosis—Professor Finlay on Alcohol in Medicine—What is Special Practice? | 79 |
| **Medical Opinion and Movement** | 80 |
| **Hospital Clinics—** | |
| Diseases of the Nervous System in Old Age. By Sir W. R. Gowers, K.C.B., M.D., F.R.S. | 81 |
| Nature's Method and the Food Supply | 83 |
| **Illustrative Cases—** | |
| Herpes Zoster and Paralysis | 85 |
| **Points in Treatment—** | |
| Diphtherial Relapses | 86 |
| **Points in Surgery—** | |
| The Treatment of Fractures from a Common-sense Point of View | 87 |
| Colostomy | 88 |
| **Otology—** | |
| Concerning Operations for Otorrhœa | 89 |
| **Practical Notes on Diagnosis and Treatment** | 90 |
| **Laryngology and Rhinology—** | |
| The Treatment of Tuberculous Laryngitis | 91 |
| **Diseases of Children—** | |
| Jaundice in the New-born | 92 |
| **The Royal Army Medical Corps Section—** | |
| The Medical Department of the New Territorial Army | 93 |
| **Dermatology—** | |
| The Value of Internal Medication in Diseases of the Skin | 94 |
| **The General Practitioner's Column—** | |
| The Advantages to be Gained by the Administration of Morphia in Certain Conditions | 95 |
| **Resident Medical Officers' Department—** | |
| Hints to Junior House Surgeons | 96 |
| **New Appliances and Things Medical** | 96 |
| **Therapeutics—** | |
| Prescriptions and Dispensing | 97 |
| **Public Health and Hygiene—** | |
| "The Surreptitious Abolition of Compulsory Vaccination" | 98 |
| **Hospital Administration—** | |
| The American Hospital Association | 99 |
| News and Coming Events | 100 |
| **Nursing Administration—** | |
| The Problem of Rural Midwifery | 101 |
| **Editor's Letter-Box—** | |
| "Professional Freedom"—The London Provident Dispensaries Council | 102 |

